# Highly expressed centromere protein L indicates adverse survival and associates with immune infiltration in hepatocellular carcinoma

**DOI:** 10.18632/aging.203574

**Published:** 2021-10-04

**Authors:** Zhili Zeng, Xiao Jiang, Zhibin Pan, Ruisheng Zhou, Zhuangteng Lin, Ying Tang, Ying Cui, Enxin Zhang, Zebiao Cao

**Affiliations:** 1 The First School of Clinical Medicine, Guangzhou University of Chinese Medicine, Guangzhou, Guangdong 510405, PR China; 2 Department of Oncology, Lingnan Medical Research Center of Guangzhou University of Chinese Medicine, Guangzhou, Guangdong 510405, PR China; 3 Department of Medical Technologic, The First Affiliated Hospital of Guangzhou University of Chinese Medicine, Guangzhou, Guangdong 518000, PR China; 4 Department of Oncology, The First Affiliated Hospital of Guangzhou University of Chinese Medicine, Guangzhou, Guangdong 518000, PR China; 5 Department of Oncology, Shenzhen Hospital of Guangzhou University of Chinese Medicine, Shenzhen 518000, Guangdong, PR China; 6 Foshan Hospital of Traditional Chinese Medicine, Guangzhou University of Chinese Medicine, Foshan 528000, Guangdong, PR China; 7 Department of Psychiatry, The Third Affiliated Hospital of Guangzhou Medical University, Guangdong 510150, PR China

**Keywords:** centromere protein L, hepatocellular carcinoma, tumor immunity, prognosis, biomarker

## Abstract

Background: Hepatocellular carcinoma (HCC) is characterized by rapid progression, high recurrence rate and poor prognosis. Early prediction for the prognosis and immunotherapy efficacy is of great significance to improve the survival of HCC patients. However, there is still no reliable predictor at present. This study is aimed to explore the role of centromere protein L (CENPL) in predicting prognosis and its association with immune infiltration in HCC.

Methods: The expression of CENPL was identified through analyzing the Cancer Genome Atlas (TCGA) and Gene Expression Omnibus (GEO) data. The association between CENPL expression and clinicopathological features was investigated by the Wilcoxon signed-rank test or Kruskal Wallis test and logistic regression. The role of CENPL in prognosis was examined via Kaplan-Meier method and Log-rank test as well as univariate and multivariate Cox regression analysis. Besides, in TIMER and GEPIA database, we investigated the correlation between CENPL level and immunocyte and immunocyte markers, and the prognostic-related methylation sites in CENPL were identified by MethSurv.

Results: CENPL had a high expression in HCC samples. Increased CENPL was prominently associated with unfavorable survival, and maybe an independent prognostic factor of worse overall survival (OS), disease-specific survival (DSS), disease-free interval (DFI), progression-free interval (PFI). Additionally, CENPL expression was significantly correlated with immune cell infiltration and some markers. CENPL also contained a methylation site that was notably related to poor prognosis.

Conclusions: Elevated CENPL may be a promising prognostic marker and associate with immune infiltration in HCC.

## INTRODUCTION

Primary liver cancer is a rapidly developing and aggressive malignant tumor with high incidence rate and short survival time. According to the statistics of the International Agency for Research on Cancer on global cancer incidence and mortality in 2020, primary liver cancer is the sixth most commonly diagnosed cancer and the third leading cause of cancer death worldwide in 2020, with approximately 906,000 new cases and 830,000 deaths and it ranks the second in the mortality rate of male cancer [1]. Hepatocellular carcinoma (HCC) is the main type of primary liver cancer [2]. Although early liver cancer can be cured by surgery or liver transplantation, more than 80% of patients have no chance of surgery. Even with successful surgical excision or liver transplantation, the postoperative recurrence is still common and elusive. Especially, early recurrence and metastasis are often difficult to be detected, so the 5-year survival rate after surgical resection and liver transplantation are only 36–70% and 60–70%, respectively [3]. The majority of patients are found in the late stage or after metastasis, while the treatment in the late stage is limited and the therapeutic effect is poor. With the clinical application of immune checkpoint inhibitors (ICIs), new dawn has been brought to tumor patients, but only a few patients have experienced clinical benefits [4]. Therefore, early prediction for the occurrence and progress as well as the immunotherapy efficacy is of great significance to improve the prognosis of HCC patients and avoid unnecessary drug toxicity. However, to date, there has been no robust predictor for HCC.

Centromere protein L (CENPL) is a member of the centromere protein (CENP) family, which is necessary for normal cell division (mitosis and meiosis) [5]. We all know that centromeres are specialized DNA sequences that connect a pair of sister chromatids, and are mainly regarded as loci that guide chromosomal behavior. The kinetochore is a cell structure attached to the centromere. During mitosis, the two sister chromatids are drawn to the poles of the cell through the spindle filaments (stellar rays of the spindle) attached to the kinetochore. If the centromere or kinetochore is abnormal, the chromosomes will be randomly allocated into the daughter cells during cell division, and the chromosome number will change. The centromere is composed of more than 15 centromere-specific proteins binding together, including the CENP family [5]. In addition, the CENP family is also the basis of kinetochore assembly and function, and they determine the correct separation of chromosomes [5, 6]. Otherwise, it will lead to abnormal chromosome number and induce tumorigenesis. Studies have shown that most cancer cells are aneuploidy [7, 8]. CENPL can be combined with CENPN to identify CENPA nucleosomes, and it plays a vital role in the process of recruiting other centromere components to assemble into centromeres [9, 10]. Previous researches have reported the role of CENPA [11, 12], CENPE [13], CENPF [14] in HCC, however, there is no research on the role of CENPL in HCC and other cancers. Based on the Cancer Genome Atlas (TCGA) and Gene Expression Omnibus (GEO) databases, we analyzed the expression of CENPL in HCC, and explored the possible pathways by which abnormally expressed CENPL was involved in the occurrence and progression of HCC. Moreover, we also explored the relationship between CENPL expression and immune cell infiltration, and investigated the abnormal methylation sites of CENPL.

## MATERIALS AND METHODS

### Gene expression profiles and clinical information

Using TCGA database (https://portal.gdc.cancer.gov/repository), we obtained the gene expression data (424 cases, Workflow Type: HTSeq-FPKM) and corresponding clinical information. Samples with deficient or unclear information on important clinicopathological characteristics were excluded. In all, 374 HCC samples and 50 adjacent normal samples were brought into our research. Patient’s clinical information contained age, gender, family history, clinical stage, TNM stage, AFP, new tumor event, residual tumor, vascular invasion, postoperative ablation embolization, tumor status, histologic grade, Child-Pugh and risk factor (viral hepatitis and/or alcohol consumption). In addition, we used gene expression profiles of GSE121248 and GSE54236, which were downloaded from the GEO database, to confirm the CENPL expression in HCC. Moreover, we validated the protein level of CENPL through obtaining the corresponding immunohistochemical (IHC) images in Human Protein Atlas (HPA) database (http://www.proteinatlas.org/). HPA is an open access knowledge repository that integrates the work of many laboratories and technology platforms around the world to explore and annotate more than 20,000 human genes in detail at the protein level [15, 16]. Image-Pro Plus software (version 6.0; Media Cybernetics, Inc.) was applied to detected the mean integrated optical density (IOD) value of IHC images. The IOD value, representing the protein level of CENPL, was statistically analyzed by non-paired *T*-test in the GraphPad Prism^®^ version 8.0 software. *P* < 0.05 indicates statistical significance.

### Gene Set Enrichment Analysis (GSEA)

A computational method called Gene Set Enrichment Analysis (GSEA) can be used to decide whether an *a priori* defined set of genes has statistical significance, concordant differences between two biological states [17]. The level of the gene was recognized as a phenotype label. The number of gene set permutations was 1,000 times for each analysis. Biological pathways with *P* < 0.05 and false discovery rate (FDR) <0.05 were considered to be significantly enriched.

### Kaplan-Meier plotter analysis

The Kaplan-Meier plotter [18] (http://www.kmplot.com/analysis/index.php?p=background) was applied to analyze the correlation between CENPL expression and survival outcome in lung cancer, ovarian cancer, breast cancer and gastric cancer. Log-rank *P*-values < 0.05 represents statistical significance.

### Co-expression, GO and KEGG enrichment analyses

Coexpedia (https://www.coexpedia.org/index.php) is a unique network tool based on the human and mouse gene chip data in the GEO database. It can analyze the functional, biological, and medical correlations between genes through statistical analysis, and then construct a co-expression network [19]. It was used to find the co-expressed genes of CENPL. Further, the co-expressed genes were utilized for Gene Ontology (GO) and the Kyoto Encyclopedia of Genes and Genomes (KEGG) enrichment analysis in the Database for Annotation, Visualization and Integrated Discovery (DAVID) database (version 6.8; https://david.ncifcrf.gov/) [20]. The annotation and functional analyses of GO consist of biological process (BP), cellular component (CC) and molecular function (MF). False discovery rate (FDR) < 0.05 was set as the enrichment cut-off value.

### Immunocyte infiltration

Tumor Immune Estimation Resource (TIMER) is an online network platform for comprehensive analysis of the immunocyte infiltration abundance (http://http://timer.cistrome.org/). It can predict the immunocyte infiltration levels in over 10000 tumors from 32 tumor types, and analyze the associations between the immunocyte infiltration abundance and gene expression, survival time, and other clinicopathological factors [21]. We searched the expression of CENPL in various cancers and uncovered the correlation between CENPL expression and six main immune cell types as well as their markers in TIMER. Furthermore, we also applied TIMER’s multivariate Cox analysis to explore the effect of immune cell infiltration and CNEPL expression on survival outcome.

### GEIPA analysis

Gene Expression Profiling Interactive Analysis (GEPIA, http://gepia.cancer-pku.cn/index.html) is a network tool based on TCGA and GTEx databases. It provides customizable functions such as tumor/normal differential expression analysis, profiling according to cancer types or pathological stages, patient survival analysis, similar gene detection, correlation analysis and dimensionality reduction analysis [22]. GEPIA was employed to validate the transcriptional level of CENPL in HCC and other cancers, and to demonstrate the relationship between CENPL expression and the major markers of immune cells.

### DNA methylation

MethSurv (https://github.com/vijayachitrabio/MethSurv) is a web tool for univariate and multivariate survival analysis based on DNA methylation data from 25 different cancer types and 7,358 patients in the TCGA database [23]. Using the MethSurv, CENPL DNA methylation sites related to the survival was disclosed.

### Statistical analysis

The expression differences of CENPL in HCC samples and normal samples, HCC samples and adjacent samples were analyzed by Wilcoxon rank-sum tests and Wilcoxon signed-rank tests, respectively. The Wilcoxon signed-rank test or Kruskal Wallis test and logistic regression were used to investigate the relationship between CENPL expression and clinicopathological factors. The role of CENPL in survival was examined via Kaplan-Meier method and Log-rank test as well as univariate and multivariate Cox regression analysis. All statistical analyses were conducted on R (version 3.6.1, 2019-07-05, R Foundation, Vienna, Austria), *P* < 0.05 was set as the statistical threshold. The median expression value of CENPL was regarded as the cut-off value.

### Ethics approval and consent to participate

No ethics approval was required for this work. All utilized public data sets were generated by others who obtained ethical approval.

### Availability of data and materials

The datasets generated and/or analyzed during the current study are available in the GSE54236 (https://www.ncbi.nlm.nih.gov/geo/query/acc.cgi?acc=GSE54236), and GSE121248 (https://www.ncbi.nlm.nih.gov/geo/query/acc.cgi?acc=GSE121248) from the Gene Expression Omnibus (GEO) database (https://www.ncbi.nlm.nih.gov/geo/).

## RESULTS

### Clinicopathological features of patients

As showed in Table 1, the clinical information of 374 HCC patients was extracted from the TCGA database in August 2020. After excluding the samples with missing or unclear clinicopathological features, finally, there were 121 females and 250 males. Only 9% (*n* = 34) patients were under 40 years old and 35.0% (*n* = 112) patients had HCC family history. Besides, most patients (65.8%, *n* = 231) had risk factors, such as alcohol consumption and/or viral hepatitis. The histologic grade included 232 (63.4%) cases in G1-2 and 134 (36.6%) cases in G3-4. The clinical stage was composed of 74.1% (*n* = 257) cases in stage I-II and 25.9% (*n* = 90) cases in stage III-IV. There were 275 of 368 (74.7%) patients in T1-2 and 93 of 368 (25.3%) patients in T3-4. Only 1.6% (4 of 256), 1.5% (4 of 270), and 34.6% (109 of 315) cases were found to have lymph node metastasis, distant metastasis, and vascular invasion, respectively. The distribution of Child-Pugh involved 90.8% Child-Pugh A patients (*n* = 217) and 9.2% Child-Pugh B-C patients (*n* = 22). 52.9% (*n* = 147) patients were in AFP < 20, 23.7% (*n* = 66) in 20 ≤ AFP < 400, and 23.4% (*n* = 65) in AFP ≥ 400. 97.7% (338 of 346) and 91.9% (319 of 347) patients did not undergone radiation therapy and postoperative chemotherapy, respectively. After operation, there were 5.3% (*n* = 18) patients with residual tumor and 48.3% (*n* = 169) patients with new tumor event. Until we extracted the data, a total of 57.1% (*n* = 201) patients were in a tumor-free state.

**Table 1:** HCC patient characteristics based on TCGA.

**Clinical characteristics**		**Total**	**%**
Age at diagnosis (years)	>40	336	91.0
	≤40	34	9.0
Gender	male	250	67.6
	female	121	32.4
Family history	Yes	112	35.0
	No	208	65.0
Histologic grade	G1-G2	232	63.4
	G3-G4	134	36.6
Clinical stage	I-II	257	74.1
	III-IV	90	25.9
T	T1-T2	275	74.7
	T3-T4	93	25.3
N	N0	252	98.4
	N1	4	1.6
M	M0	266	98.5
	M1	4	1.5
Residual tumor	R0	324	94.7
	R1	18	5.3
Tumor status	tumor free	201	57.1
	with tumor	151	42.9
Vascular invasion	Yes	109	34.6
	No	206	65.4
Child-Pugh	A	217	90.8
	B-C	22	9.2
AFP	AFP <20	147	52.9
	20< AFP <400	66	23.7
	AFP ≥400	65	23.4
New tumor event	Yes	169	48.3
	No	181	51.7
Risk factor	Alcohol consumption and viral hepatitis	39	11.1
	Alcohol consumption	78	22.2
	Viral hepatitis	114	32.5
	Neither	120	34.2
Postoperative ablation embolization	Yes	28	8.1
	No	319	91.9
Radiation therapy	Yes	8	2.3
	No	338	97.7

Abbreviations: T: topography distribution; N: lymph node metastasis; M: distant metastasis; AFP: alpha fetal protein.

### The expression of CENPL in HCC and other cancers

**Figure 1:**
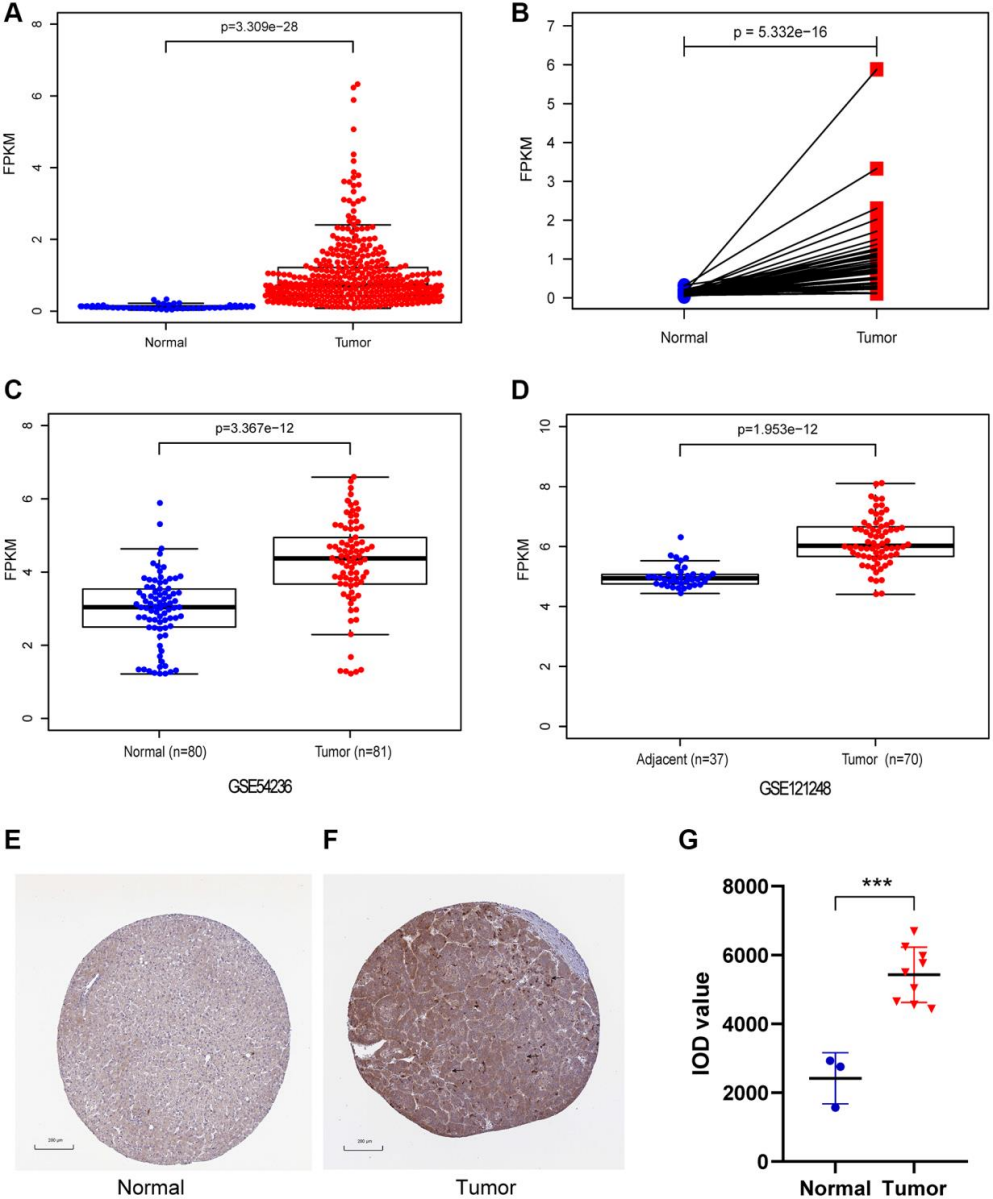
**The mRNA and protein expression of CENPL in HCC.** (**A**) CENPL showed significantly high expression in HCC tissues than in normal tissues using Wilcoxon rank sum test. (**B**) The expression of CENPL was prominently highly expressed in HCC tissues compared with adjoining non-cancerous tissues via Wilcoxon signed-rank test. (**C** and **D**) suggested CENPL was prominently enhanced in HCC samples from GSE121248 and GSE54236. (**E**–**G**) compared with the normal group, the protein level of CENPL in the liver cancer group was significantly increased. Abbreviation: CENP: centromere protein.

In our study, CENPL expression was compared between 374 HCC samples and 50 adjacent normal samples. We found that the expression of CENPL was significantly elevated in HCC (Figure 1A, 1B). This result was confirmed in GSE121248 and GSE54236 datasets (Figure 1C, 1D). Further, the TIMER and GEPIA analysis showed that the transcriptional levels of CENPL in various cancer types, such as HCC, lung cancer, pancreatic cancer, ovarian cancer, breast cancer, gallbladder cancer, cervical cancer, esophageal cancer, glioblastoma multiforme Tumor, bladder urothelial carcinoma, were significantly higher than that in normal tissues (Supplementary Figures 1 and 2). Moreover, we found that the protein level of CENPL was also significantly increased in HCC based on the HPA database (Figure 1E–1G). These results suggested that high mRNA and protein levels of CENPL in HCC were consistent in different databases.

### Association between CENPL and clinicopathological factors

**Figure 2:**
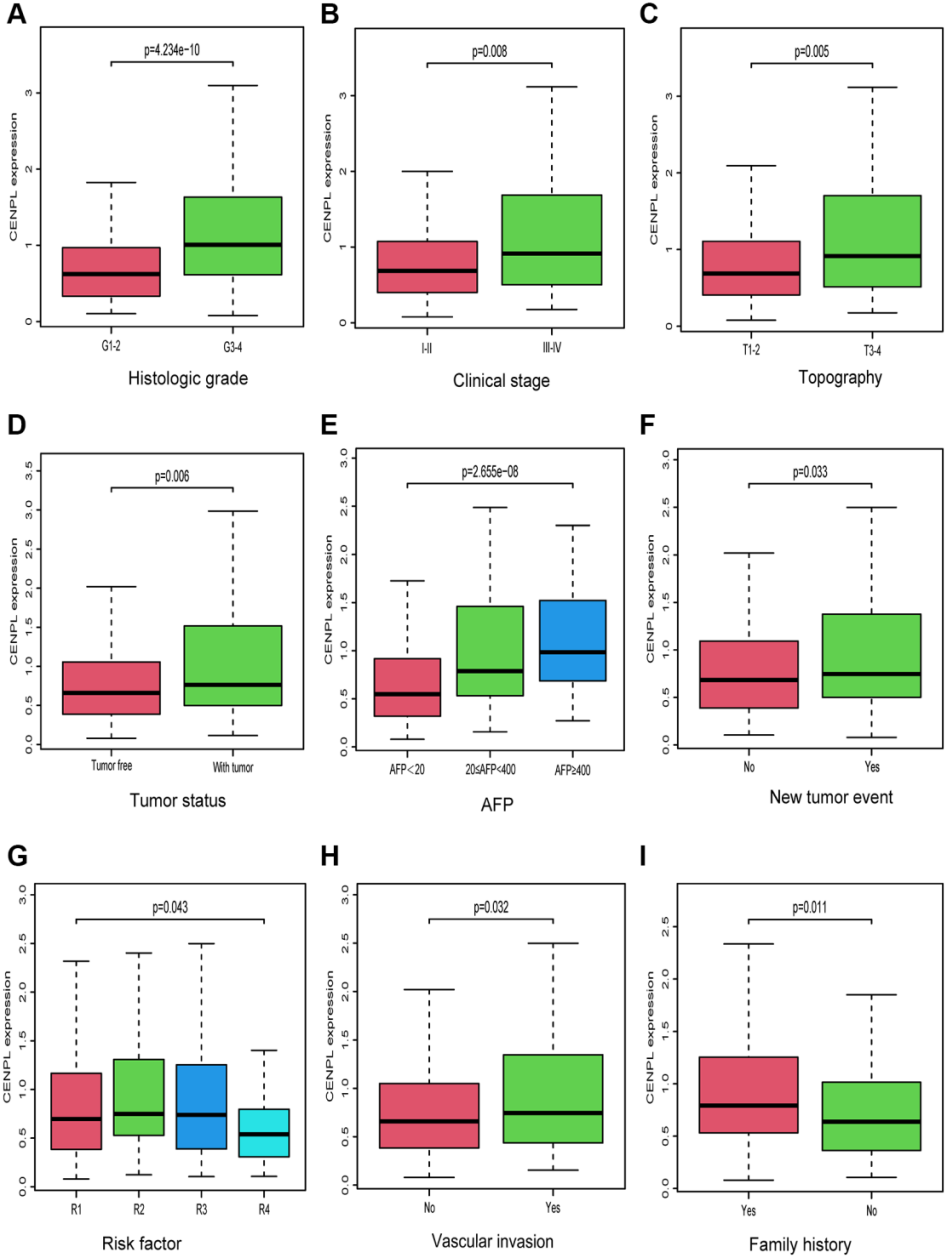
**Relationship between CENPL transcription level and clinicopathologic characteristics.** Increased CENPL had significant correlation with (**A**) histologic grade, (**B**) clinical stage, (**C**) topography, (**D**) tumor status, (**E**) AFP, (**F**) New tumor event, (**G**) Risk factor, (**H**) Vascular invasion, (**I**) Family history. Abbreviations: CENP: centromere protein; T: topography distribution; N: lymph node metastasis; M: distant metastasis; AFP: alpha fetal protein.

As we can see in Figure 2A–2I, up-regulated CENPL was distributed in patients with family history (Yes vs. No, *p* = 0.011), vascular invasion (No vs. Yes, *p* = 0.032), new tumor event (*p* = 0.033), risk factor (R1 vs. R2 vs. R3 vs. R4, *p* = 0.043), with tumor (*p* = 0.006), high AFP (AFP < 20 vs. 20 ≤ AFP < 400 vs. AFP ≥ 400, *p* = 2.655e−08) as well as unfavorable histologic grade (G1-2 vs. G3-4, *p* = 4.234e−10), clinical stage (Stage I−II vs. Stage III−IV, *p* = 0.008) and topography (T) stage (T1-2 vs. T3-4, *p* = 0.005). Similar but more specific results were obtained by logistic regression analysis (Table 2).

**Table 2:** Association between CENPL expression and clinicopathologic characteristics (logistic regression).

**Clinical characteristics**	**Total**	**OR**	***P*-value**
Age at diagnosis (years) (>40 vs. ≤40)	370	0.9 (0.4–1.8)	0.719
Gender (female vs. male)	371	0.6 (0.4–1.0)	**0.033**
Family history (no vs. yes)	320	0.6 (0.4–0.9)	**0.020**
Histologic grade (G3-4 vs. G1-2)	366	3.4 (2.2–5.4)	**0.000**
Clinical stage (III-IV vs. I-II)	347	1.7 (1.1–2.8)	**0.026**
T (T3-4 vs. T1-2)	368	1.8 (1.1–3.0)	**0.012**
N (N1 vs. N0)	256	1.0 (0.1–8.4)	1.000
M (M1 vs. M0)	270	1.0 (0.1–8.4)	1.000
Residual tumor (R1-2 vs. R0)	342	1.6 (0.6–4.5)	0.337
Tumor status (with tumor vs. tumor free)	352	1.6 (1.0–2.4)	**0.041**
Vascular invasion (yes vs. no)	315	1.5 (1.0–2.5)	0.070
Child-Pugh (B-C vs. A)	239	1.6 (0.6–4.5)	0.337
New tumor event (yes vs. no)	350	1.4 (0.9–2.2)	0.109
Radiation therapy (yes vs. no)	346	0.6 (0.1–2.5)	0.479
Postoperative ablation embolization (yes vs. no)	347	1.2 (0.5–2.6)	0.682
Risk factor			
RF2 vs. RF1	235	1.3 (0.8–2.2)	0.319
RF3 vs. RF1	199	1.2 (0.7–2.1)	0.600
RF4 vs. RF1	160	0.5 (0.2–1.1)	0.095
RF3 vs. RF2	192	0.9 (0.5–1.6)	0.712
RF4 vs. RF2	153	0.4 (0.2–0.9)	**0.020**
RF4 vs. RF3	117	0.5 (0.2–1.0)	0.051
AFP			
AFP2 vs. AFP1	213	2.9 (1.6–5.4)	**0.000**
AFP3 vs. AFP1	131	4.0 (2.2–7.6)	**0.000**
AFP3 vs. AFP2	212	1.4 (0.7–2.9)	0.392

Abbreviations: OR: odd ratio; T: topography distribution; N: lymph node metastasis; M: distant metastasis; RF: risk factor (RF1: neither alcohol consumption or viral hepatitis; RF2: viral hepatitis; RF3: alcohol consumption; RF4: both alcohol consumption and viral hepatitis); AFP: alpha fetal protein (AFP1 represents AFP <20; AFP2 represents 20≤ AFP <400; AFP3 represents AFP ≥400).

### The prognostic value of enhanced CENPL

**Figure 3:**
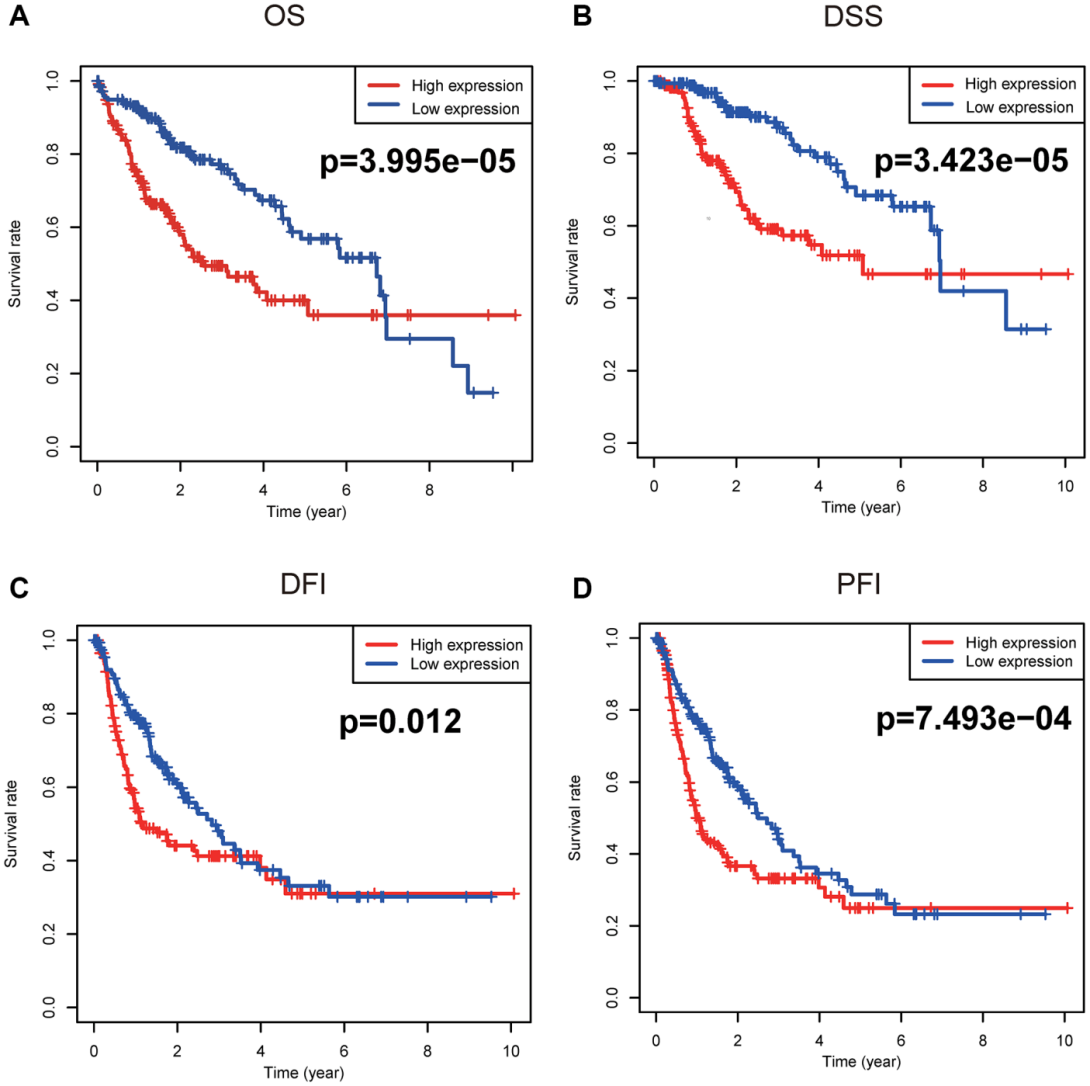
**Survival analyses based on Kaplan-Meier method.** Kaplan-Meier survival analysis indicated that elevated CENPL was prominently associated with worse (**A**) OS, (**B**) DSS, (**C**) DFI, and (**D**) PFI. Abbreviations: CENP: centromere protein; OS: overall survival; DSS: disease-specific survival; DFI: disease-free interval; PFI: progression-free interval.

**Figure 4:**
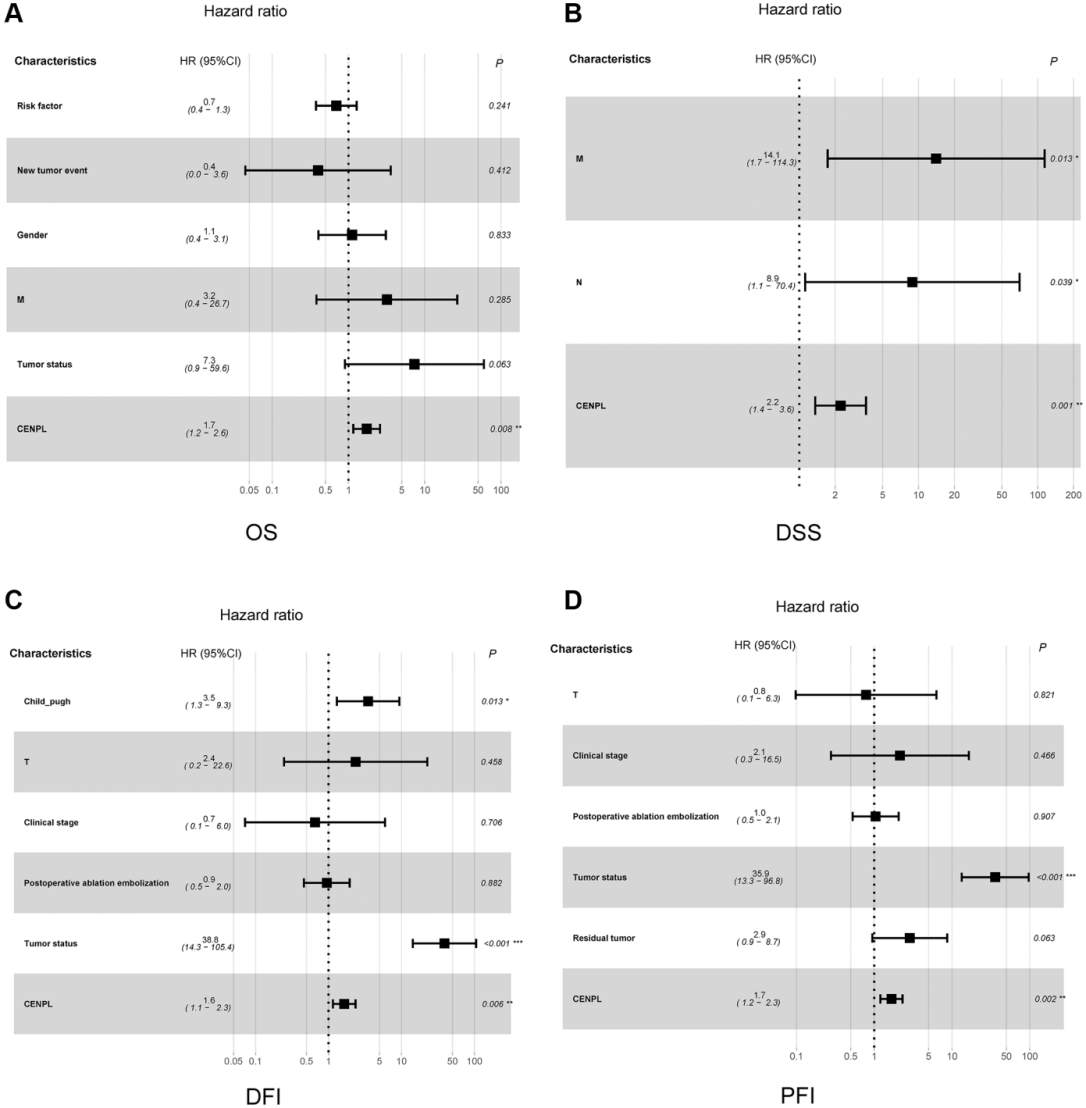
**Relationship between clinicopathologic factors and survival outcome of HCC patient through univariate and multivariate Cox regression analysis.** (**A**–**D**) indicated CENPL may be an independent prognostic factor for poor OS, DSS, DFI and PFI, respectively. Abbreviations: OS: overall survival; DSS: disease-specific survival; DFI: disease-free interval; PFI: progression-free interval.

Figure 3 unveiled that enhanced CENPL was significantly related to poor overall survival (OS) (*p* = 3.995e−05), disease-specific survival (DSS) (*p* = 3.423e−05), disease-free interval (DFI) (*p* = 0.012), and progression-free interval (PFI) (*p* = 7.493e−04). Moreover, this similar phenomenon occurred in other cancers, such as OS, post progression survival (PPS), first progression (FP) in lung cancer; OS, PPS, progression-free survival (PFS) in ovarian cancer; OS, relapse-free survival (RFS), distance metastasis free survival (DMFS) in breast cancer (Supplementary Figure 3). Furthermore, univariate and multivariate Cox regression analysis found that elevated CENPL can independently predict worse OS (HR = 1.7, 95% CI [1.2–2.6], *p* = 0.008), DSS (HR = 2.2, 95% CI [1.4–3.6], *p* = 0.001), DFI (HR = 1.6, 95% CI [1.1–2.3], *p* = 0.006), and PFI (HR = 1.7, 95% CI [1.2–2.3], *p* = 0.002) in HCC (Table 3, Figure 4).

**Table 3:** Association between clinicopathologic characteristics and OS, DSS, DFI and PFI in patients with HCC through univariate and multivariate analysis with Cox regression survival model.

**Characteristics**	**OS**			**DSS**			**DFI**			**PFI**		
	**HR**	**95% CI**	***P*-value**	**HR**	**95% CI**	***P*-value**	**HR**	**95% CI**	***P*-value**	**HR**	**95% CI**	***P*-value**
**Univariate analysis**												
Child-Pugh (B-C vs. A)	1.3	0.4–4.2	0.715	1.8	0.5–6.3	0.363	2.1	0.9–4.9	0.096	1.8	0.8–4.3	0.164
Risk factor (Alcohol consumption and/or viral hepatitis vs. neither)	0.6	0.4–1.0	0.064	0.6	0.3–1.1	0.12	0.9	0.6–1.2	0.341	0.8	0.6–1.1	0.244
AFP (20≤ AFP vs. AFP <20)	1.0	0.7–1.6	0.961	1.0	0.6–1.6	0.871	0.9	0.7–1.3	0.632	1.0	0.7–1.3	0.914
New tumor event (yes vs. no)	3.0	1.3–7.0	**0.012**	1.4	0.9–2.1	0.997	1.0	0.8–1.5	0.996	4.3	1.9–10.1	0.995
Age (>40 vs. ≤40)	4.7	0.6–34.8	0.133	3.8	0.5–28.7	0.197	1.0	0.4–2.3	0.926	0.9	0.4–2.1	0.887
Gender (male vs. female)	0.5	0.2–1.1	0.086	0.6	0.2–1.3	0.195	0.9	0.5–1.6	0.599	0.8	0.5–1.4	0.406
Histologic grade (G3-4 vs. G1-2)	1.6	0.7-3.3	0.247	1.5	0.6–3.6	0.35	1.2	0.7–2.1	0.486	1.1	0.7–1.9	0.645
M (M1 vs. M0)	5.4	0.7–40.7	0.099	8.9	1.1–68.6	**0.037**	1.1	0.9–1.4	1.000	5.0	0.7–37.4	0.116
N (N1 vs. N0)	4.3	0.6–31.7	0.157	6.1	0.8–46.7	0.081	4.2	0.6–31.4	0.159	3.7	0.5–27.5	0.197
T (T3-4 vs. T1-2)	1.2	0.5–3.0	0.691	1.8	0.7–4.6	0.25	2.0	1.0–3.9	**0.043**	1.9	1.0–3.6	**0.047**
Clinical stage (III-IV vs. I-II)	1.4	0.6–3.3	0.454	2.1	0.8–5.2	0.117	2.1	1.1–4.1	**0.022**	2.0	1.1–3.8	**0.026**
Postoperative ablation embolization (yes vs. no)	1.1	0.4–3.2	0.87	1.5	0.5–4.6	0.47	2.7	1.4–5.5	**0.005**	2.7	1.4–5.1	**0.003**
Radiation therapy (yes vs. no)	0.6	0.3–1.2	0.997	1.3	0.8–2.0	0.997	1.5	0.2–11.1	0.683	1.3	0.2–9.6	0.787
Vascular invasion (yes vs. no)	1.3	0.6–2.9	0.560	1.1	0.4–3.1	0.821	1.0	0.5–2.0	0.910	1.2	0.7–2.1	0.590
Tumor status (with tumor vs. tumor free)	4.0	1.8–9.2	**0.001**	2.2	1.0–4.9	0.997	35.0	13.4–93.5	**0.000**	37.0	14.2–97.4	**0.000**
Family history (yes vs. no)	1.8	0.9–3.7	0.116	2.0	0.8–4.7	0.119	1.1	0.6–2.1	0.639	1.1	0.6–1.9	0.785
Residual tumor (R1-2 vs. R0)	1.4	0.2–10.2	0.761	2.3	0.3–17.4	0.434	1.3	1.1–1.5	0.774	6.4	2.2–18.3	**0.001**
CENPL (high vs. low)	1.8	1.2–2.6	**0.002**	2.0	1.3–3.3	**0.003**	1.9	1.3–2.6	**0.000**	1.8	1.3–2.5	**0.000**
**Multivariate analysis**												
Child-Pugh (B-C vs. A)							3.5	1.3–9.3	**0.013**			
Risk factor (Alcohol consumption and/or viral hepatitis vs. neither)	0.7	0.4–1.3	0.241									
New tumor event (yes vs. no)	0.4	0.0–3.6	0.412									
Gender (male vs. female)	1.1	0.4–3.1	0.833									
M (M1 vs. M0)	3.2	0.4–26.7	0.285	14.0	1.7–114.3	**0.013**						
N (N1 vs. N0)				8.9	1.1–70.4	**0.039**						
T (T3-4 vs. T1-2)							2.4	0.2–22.6	0.458	0.8	0.1–6.3	0.821
Clinical stage (III-IV vs. I-II)							0.7	0.1–6.0	0.706	2.1	0.3–16.5	0.466
Postoperative ablation embolization (yes vs. no)							0.9	0.5–2.0	0.882	1.0	0.5–2.1	0.907
Tumor status (with tumor vs. tumor free)	7.3	0.9–59.6	0.063				39.0	14.3–105.4	**0.000**	36.0	13.3–96.8	**0.000**
Residual tumor (R1-2 vs. R0)										2.9	0.9–8.7	0.063
CENPL (high vs. low)	1.7	1.2–2.6	**0.008**	2.2	1.4–3.6	**0.001**	1.6	1.1–2.3	**0.006**	1.7	1.2–2.3	**0.002**

Abbreviations: OS: overall survival; DSS: disease-specific survival; DFI: disease-free interval; PFI: progression free interval; T: topography distribution; N: lymph node metastasis; M: distant metastasis; HR: hazard ratio; CI: confidence interval; CENPL: centromere protein L.

### Pathways related to CENPL in HCC

**Figure 5:**
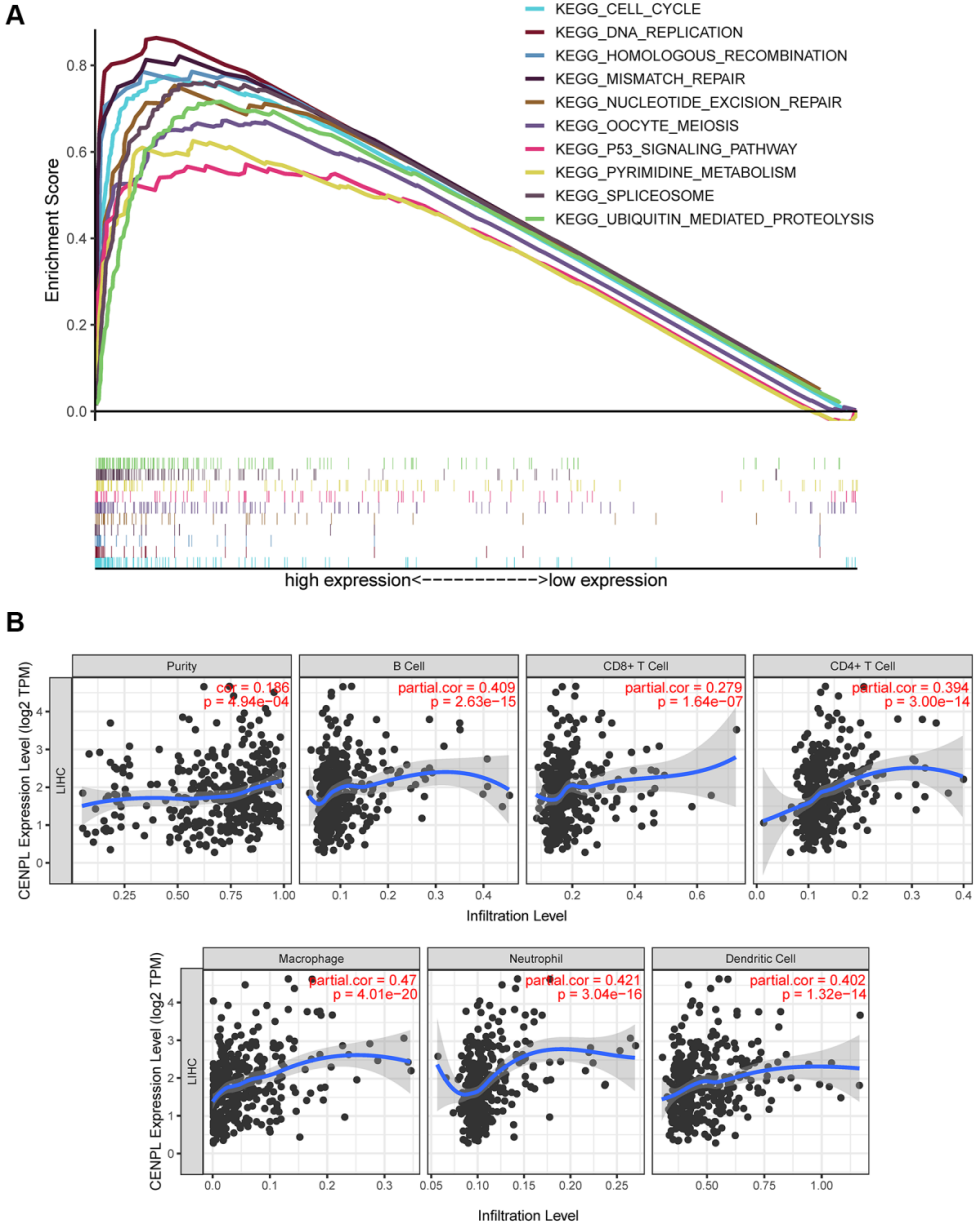
(**A**) Enrichment plots from GSEA. GSEA results showed that spliceosome, nucleotide excision repair, pyrimidine metabolism, DNA replication, ubiquitin mediated proteolysis, cell cycle, homologous recombination, mismatch repair, p53 signaling pathway and oocyte meiosis were significantly enriched in the high CENPL expression phenotype. GSEA: Gene Set Enrichment Analysis. (**B**) CENPL transcription level had prominent association with level of immune cell infiltration in HCC.

GSEA unraveled that spliceosome, nucleotide excision repair, DNA replication, cell cycle, homologous recombination, ubiquitin mediated proteolysis, mismatch repair, p53 signaling pathway, oocyte meiosis and pyrimidine metabolism were significantly enriched in the high CENPL expression phenotype (Table 4; Figure 5A), indicating that elevated CENPL might participate in the occurrence and progression of HCC through these pathways.

**Table 4:** Gene sets enriched in phenotype high.

**MSigDB collection**	**Gene set name**	**NES**	**NOM** ***p*-value**	**FDR** ***q*-value**
c2.cp.kegg. v7.0.symbols. gmt [Curated]	KEGG_UBIQUITIN_MEDIATED_PROTEOLYSIS	2.177	0.000	0.000
	KEGG_CELL_CYCLE	2.132	0.000	0.002
	KEGG_OOCYTE_MEIOSIS	2.128	0.000	0.001
	KEGG_PYRIMIDINE_METABOLISM	2.057	0.000	0.003
	KEGG_NUCLEOTIDE_EXCISION_REPAIR	2.056	0.000	0.002
	KEGG_SPLICEOSOME	1.981	0.002	0.004
	KEGG_DNA_REPLICATION	1.968	0.000	0.004
	KEGG_HOMOLOGOUS_RECOMBINATION	1.940	0.000	0.004
	KEGG_MISMATCH_REPAIR	1.921	0.000	0.004
	KEGG_P53_SIGNALING_PATHWAY	1.884	0.000	0.006

Abbreviations: NES: normalized enrichment score; NOM: nominal; FDR: false discovery rate. Gene sets with NOM *p*-value <0.05 and FDR *q*-value <0.05 are considered as significant.

**Figure 6:**
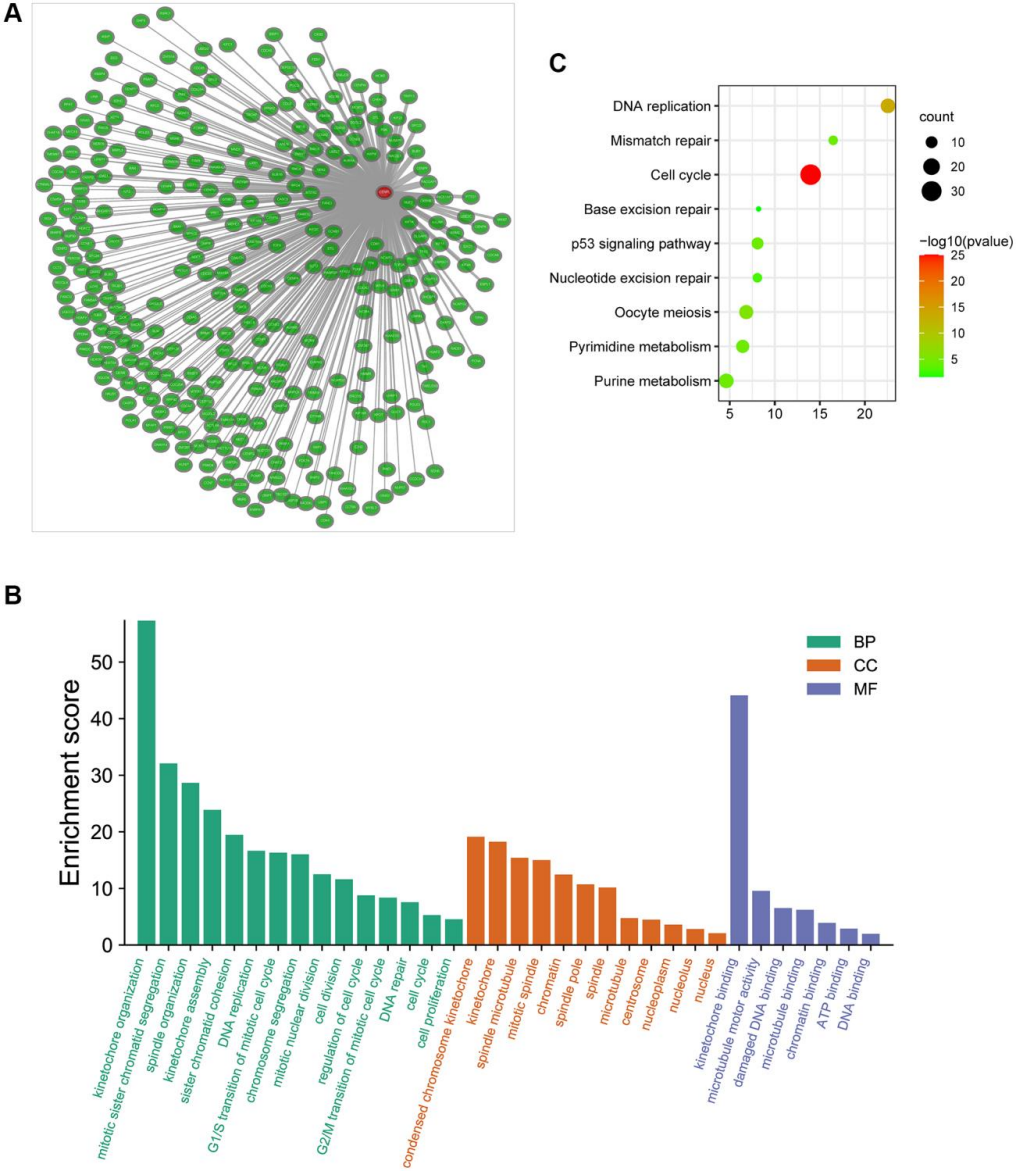
(**A**) The co-expression network of CENPL; (**B**–**C**) GO and KEGG enrichment analysis of co-expression genes of CENPL. Abbreviations: CENPL: centromere protein L; GO: Gene Ontology; KEGG: Kyoto Encyclopedia of Genes and Genomes.

Additionally, we obtained the co-expressed genes of CENPL in Coexpedia database (Figure 6A). Among these genes, Nuf2 had the strongest positive correlation with CENPL in HCC and many other common cancers (Supplementary Figure 4). Since co-expressed genes often have similar functions [24], we analyzed the enrichment pathways of these co-expressed genes via DAVID. For BP, co-expression genes were significantly enriched in kinetochore organization, mitotic sister chromatid segregation, spindle organization, kinetochore assembly, sister chromatid cohesion DNA replication. For CC, co-expression genes were prominently enriched in condensed chromosome kinetochore, kinetochore, spindle microtubule, mitotic spindle, chromatin. For MF, co-expression genes were significantly enriched in kinetochore binding, microtubule motor activity, damaged DNA binding, microtubule binding, chromatin binding, ATP binding and DNA binding (Figure 6B). KEGG pathway enrichment analysis showed that co-expression genes were significantly enriched in cell cycle, DNA replication, oocyte meiosis, pyrimidine metabolism, mismatch repair, p53 signaling pathway, purine metabolism, nucleotide excision repair and base excision repair (Figure 6C).

### The effect of CENPL expression on immune cell infiltration

Based on the TIMER and GEPIA database, we found that CENPL expression was positively correlated with the infiltration of B cell (r = 0.409, *p* = 2.63e−15), CD8+ T Cell (r = 0.279, *p* = 1.64e−07), CD4+ T Cell (r = 0.394, *p* = 3.00e−14), Macrophage (r = 0.47, *p* = 4.01e−20), Neutrophil (r = 0.421, *p* = 3.04e−16), Dendritic Cell (r = 0.402, *p* = 1.32e−14) (Figure 5B), as well as some immunocyte biomarkers (Supplementary Figure 5 and Table 5). In addition, TIMER multivariate Cox analysis exhibited that B cell (Coef = −7.514, HR = 0.00, *p* = 0.045), CD8 T cell (Coef = −5.056, HR = 0.01, *p* = 0.046), CD4 T cell (Coef = −9.241, HR = 0.00, *p* = 0.016) infiltration were negative independent predictors for poor prognosis, while macrophage (Coef = 8.546, HR = 5146.34, *p* = 0.003) and dendritic (Coef = 4.945, HR = 140.42, *p* = 0.012) infiltration and CENPL expression were positive independent predictors for poor prognosis (Table 6), indicating low B cell, CD8 T cell, CD4 T cell infiltration and high macrophage and dendritic infiltration as well as CENPL expression predicted poor prognosis.

**Table 5:** Survival analysis of multivariate COX hazard model based on TIMER online tool.

**Clinicopathologic variable**	**Coef**	**95% CI**	**HR**	***P*-value**
Age	0.016	1.00−1.03	1.02	0.068
Gender Male	−0.019	0.61−1.59	0.98	0.938
Race Black	1.201	1.20−9.21	3.32	**0.021**
Race White	−0.002	0.60−1.66	1.00	0.995
Stage II	0.081	0.63−1.87	1.09	0.770
Stage III	0.748	1.30−3.43	2.11	**0.003**
Stage IV	1.522	1.31−15.99	4.58	**0.017**
Purity	0.419	0.47−4.95	1.52	0.487
B cell	−7.514	0.00−0.84	0.00	**0.045**
CD8 T cell	−5.056	0.00−0.92	0.01	**0.046**
CD4 T cell	−9.241	0.00−0.18	0.00	**0.016**
Macrophage	8.546	18.24−1452349.07	5146.34	**0.003**
Neutrophil	−5.479	0.00−478.27	0.00	0.357
Dendritic	4.945	3.02−6526.97	140.42	**0.012**
CENPL	0.389	1.11−1.96	1.48	**0.007**

Abbreviations: TIMER: Tumor Immune Estimation Resource; CENPL: centromere protein L; HR: hazard ratio; Coef: coefficient.

**Table 6:** The association between the expression of CENPL and immune biomarker genes in HCC based on TIMER and GEPIA.

**Description**	**Gene markers**	**TIMER**		**GEPIA**	
		**Cor**	***P*-value**	**Cor**	***P*-value**
CD8+ T cell	CD8A	0.284	**8.18E−08**	0.160	**2.30E-03**
	CD8B	0.209	**8.84E−05**	0.093	**7.50E-02**
T cell (general)	CD2	0.282	**1.03E−07**	0.130	**1.40E-02**
	CD3E	0.282	**9.94E−08**	0.120	**2.30E-02**
	CD3D	0.288	**4.89E−08**	0.120	**1.70E-02**
	CD6	0.259	**1.04E−06**	0.130	**1.20E-02**
	SH2D1A	0.271	**3.36E−07**	0.140	**8.00E-03**
	TRAT1	0.263	**7.37E−07**	0.150	**3.40E-03**
	CD3G	0.323	**7.76E−10**	0.250	**8.2E−07**
B cell	CD19	0.293	**2.96E−08**	0.230	**1.2E−05**
	CD79A	0.241	**5.93E−06**	0.120	**2.50E-02**
Monocyte	CD86	0.412	**1.50E−15**	0.270	**2.4E−07**
	CD115 (CSF1R)	0.295	**2.22E−08**	0.180	**4.90E-04**
TAM	CD68	0.282	**9.54E−08**	0.200	**9.9E−05**
	CCL2	0.216	**5.04E−05**	0.110	**4.30E-02**
	IL10	0.284	**7.94E−08**	0.150	**4.60E-03**
M1 Macrophage	IRF5	0.478	**3.86E−21**	0.440	**1.1E−18**
M2 Macrophage	CD163	0.194	**2.91E−04**	−0.006	9.10E**−**01
	VSIG4	0.238	**8.12E−06**	0.120	**2.60E−02**
	MS4A4A	0.228	**1.99E−05**	0.100	5.10E**−**02
Neutrophil	S100A12	0.000	9.99E−01	−0.023	6.60E**−**01
	CEACAM3	0.159	**3.10E−03**	0.120	**2.20E−02**
	CCR7	0.229	**1.69E−05**	0.100	5.30E**−**02
	FPR1	0.322	**9.78E−10**	0.200	**7.4E−05**
	SIGLEC5	0.415	**7.99E−16**	0.240	**2.6E−06**
	CSF3R	0.375	**5.58E−13**	0.240	**3.9E−06**
	FCAR	0.196	**2.42E−04**	0.094	7.20E**−**02
	FCGR3B	0.232	**1.33E−05**	0.320	**3.1E−10**
NK	KIR2DL1	−0.051	3.41E−01	0.056	2.80E**−**01
	KIR2DL3	0.253	**1.89E−06**	0.200	**1.50E−04**
	KIR2DL4	0.184	**5.92E−04**	0.200	**1E−04**
	KIR3DL1	0.039	4.75E−01	−0.048	3.60E**−**01
	KIR3DL2	0.170	**1.54E−03**	0.200	**7.9E−05**
	KIR3DL3	0.186	**4.94E−04**	0.120	**1.90E−02**
	XCL1	0.304	**8.72E−09**	0.240	**2.5E−06**
	XCL2	0.245	**3.98E−06**	0.082	1.20E**−**01
	NCR1	0.119	**2.69E−02**	0.140	**5.90E−03**
DC	CD11C (ITGAX)	0.407	**3.11E−15**	0.300	**7.5E−09**
	HLA-DPA1	0.280	**1.25E−07**	0.160	**1.70E−03**
	HLA-DRA	0.309	**4.38E−09**	0.190	**2.10E−04**
	HLA-DQB1	0.204	**1.33E−04**	0.042	4.20E**−**01
	HLA-DPB1	0.274	**2.23E−07**	0.160	**1.60E−03**
	CCL13	0.162	**2.59E−03**	0.090	8.30E**−**02
	HSD11B1	−0.234	**1.17E−05**	−0.2	**9.5E−05**
Th1	TBX21 (T-bet)	0.171	**1.39E−03**	0.072	1.70E**−**01
	TNF	0.334	**1.92E−10**	0.230	**1.2E−05**
	STAT1	0.434	**2.76E−17**	0.390	**7.2E−15**
	STAT4	0.296	**2.20E−08**	0.240	**3.1E−06**
Th2	IL13	0.107	**4.79E−02**	0.130	**1.10E-02**
	GATA3	0.326	**5.59E−10**	0.210	**3.6E−05**
	STAT5A	0.386	**1.04E−13**	0.340	**2.1E−11**
	STAT6	0.231	**1.50E−05**	0.280	**3.3E−08**
Tfh	VSIG4	0.241	**5.98E−06**	0.120	**2.60E-02**
Th17	STAT3	0.278	**1.59E−07**	0.270	**9.4E−08**
Treg	TGFB1	0.366	**2.32E−12**	0.220	**1.6E−05**
	STAT5B	0.379	**3.29E−13**	0.430	**2.4E−18**
	CCR8	0.504	**1.20E−23**	0.410	**2.7E−16**
	FOXP3	0.264	**6.30E−07**	0.140	**5.50E-03**
T-cell exhaustion	TIGIT	0.367	**1.89E−12**	0.230	**7.2E−06**
	GZMB	0.137	**1.10E−02**	0.019	7.20E**−**01
	TOX	0.322	**9.49E−10**	0.240	**5E−06**
	TIM-3 (HAVCR2)	0.414	**9.94E−16**	0.250	**7.3E−07**
	LAG3	0.278	**1.46E−07**	0.140	**6.90E−03**
	CTLA4	0.364	**3.02E−12**	0.240	**2.5E−06**
	PD-1 (PDCD1)	0.363	**3.60E−12**	0.240	**2.6E−06**

Abbreviations: TAM: tumor-associated macrophage; IL: interleukin; Tfh: follicular helper T cell; TNF: tumor necrosis factor; DC: dendritic cell; Treg: regulatory T cell; TIMER: Tumor Immune Estimation Resource; GEPIA: Gene Expression Profiling Interactive Analysis; NK: natural killer cell; Th: T helper cell; HCC: hepatocellular carcinoma; Cor: R value of Spearman’s correlation; CENPL: centromere protein L.

In TIMER database, CENPL expression is prominently positively correlated with markers of M1 Macrophage (IRF5: Cor = 0.478, *P* = 3.86E−21), M2 Macrophage (CD163: Cor = 0.194, *P* = 2.91E−04; VSIG4: Cor = 0.238, *P* = 8.12E−06; MS4A4A: Cor = 0.228, *P* = 1.99E−05), Treg (STAT5B: Cor = 0.379, *P* = 3.29E−13; CCR8: Cor = 0.504, *P* = 1.20E−23), B cell and T cell, especially T-cell failure markers such as PD-1(Cor = 0.363, *P* = 3.60E−12), CTLA4 (Cor = 0.364, *P* = 3.02E−12), LAG3 (Cor = 0.278, *P* = 1.46E−07), TOX (Cor = 0.322, *P* = 9.49E−10), TIGIT (Cor = 0.367, *P* = 1.89E−12), GZMB (Cor = 0.137, *P* = 1.10E−02) and Tim-3 (Cor = 0.414, *P* = 9.94E−16). Similar results were obtained in GEPIA database.

### The methylation site of CENPL

**Figure 7:**
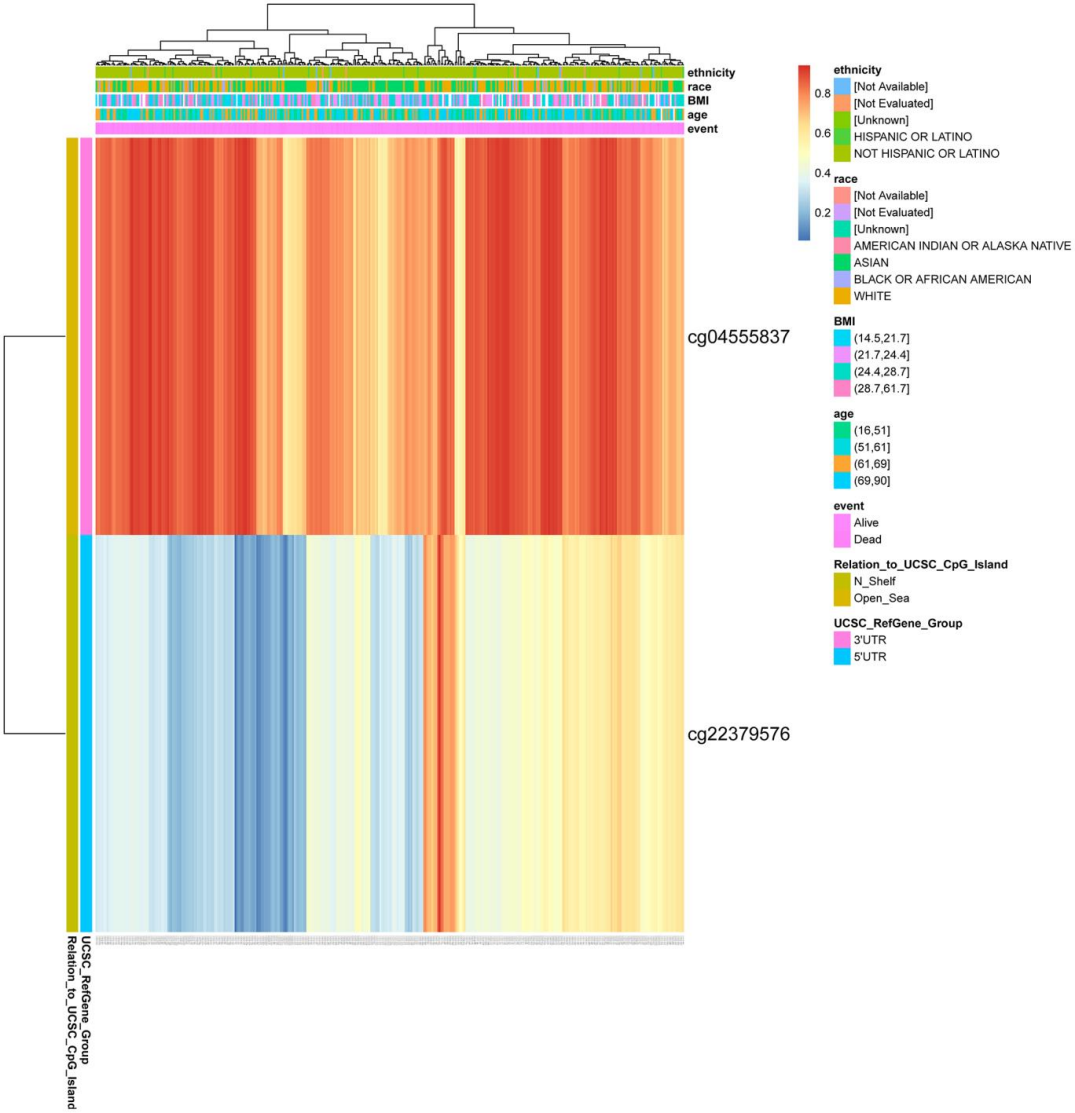
**DNA methylation of CENPL.** Red to blue means that the expression level goes from high to low. The different colored boxes represent ethnicity, race, BMI, age, event, relation_to_UCSC_CpG_Island, UCSC_RefGene_Group. Abbreviations: CENPL: centromere protein L; BMI: Body Mass Index.

We found two DNA methylation sites of CENPL, namely, CENPL-3′UTR−Open_Sea−cg04555837 and CENPL-5′UTR−N_Shelf−cg22379576 using MethSurv web tool. Among them, CENPL-3′UTR−Open_Sea−cg04555837 was significantly positively related to shorter survival time, with a HR of 2.431 (Table 7; Figure 7).

**Table 7:** CpG of CENPL related to the prognosis of HCC.

**Gene-CpG**	**HR**	**LR test *p* value**
CENPL-3′UTR−Open_Sea−cg04555837	2.431	**0.000**
CENPL-5′UTR−N_Shelf−cg22379576	1.232	0.232

Abbreviations: CENPL: centromere protein L; HR: hazard ratio; LR: log-rank.

## DISCUSSION

Although breakthroughs have been made in various diagnosis and treatment methods, including biomarkers and immunotherapy, the prognosis of HCC patients is still not optimistic [1]. Therefore, finding markers that can not only predict the occurrence, development and prognosis of HCC, but also predict the effect of immunotherapy is contributed to correctly diagnosing and intervening HCC in the early stage, improving the prognosis, and reducing unnecessary adverse drug reactions.

Through integrating multiple databases, we found that CNEPL was enhanced in most cancer types, which indicated that CENPL had a wide range of applicability and functional conservation. However, CENPL was decreased in Acute Myeloid Leukemia (LAML) and testicular germ cell tumor (TGCT) in GEPIA, which indicated that the transcription level of CENPL was still specific in different tumors. We further found that high expression of CENPL was significantly associated with adverse clinicopathological characteristics. Survival analysis revealed that patients with high CENPL expression had worse OS, DSS, DFI, and PFI. Most importantly, univariate and multivariate Cox regression analysis proved that elevated CENPL was an independent risk factor for poor OS, DSS, DFI and PFI in HCC patients. GSEA results indicated that CENPL may be involved in the occurrence and progression of HCC via some pathways. Among these pathways, cell cycle, DNA replication, p53 signaling pathway, and oocyte meiosis play important roles in regulating cell cycle. The hallmarks of cancer comprise sustaining proliferative signaling, evading growth suppressors, resisting cell death, enabling replicative immortality, inducing angiogenesis, and activating invasion and metastasis [25], and these processes all involve cell cycle abnormalities. Nucleotide excision repair [26, 27], mismatch repair [28], homologous recombination [29] are key pathways for repairing DNA damage and preventing tumorigenesis. Disorders in these pathways could lead to genetic mutations, chromosomal aberrations, and subsequent transcriptional and translation errors, which gradually accumulate and result in cancer [26]. Pyrimidine metabolism, a branch of nucleotide metabolism, provides pyrimidine base nucleotides and deoxyribonucleotides to synthetize DNA and RNA, which plays a crucial role in maintaining basic cellular functions [30, 31]. Dysfunctional pyrimidine metabolism can facilitate cancer proliferation and invasiveness and induce HCC epithelial-mesenchymal transition (EMT), thereby enhancing the stem cell-like characteristics and drug resistance of cancer cells [31–33]. Studies have suggested that pyrimidine metabolism pathways are promising targets for HCC treatment [30, 34]. In addition, spliceosomes exert a key effect in the process of removing introns, connecting exons on both sides and transforming into mature mRNAs after transcription [35–37]. Pre-mRNA splicing is a key step in gene expression [38]. Abnormal RNA spliceosomes and/or splicing processes have been shown to promote tumor genesis and maintenance in a variety of ways [39–41], and some laboratories have begun to develop and design spliceosome inhibitors for anti-tumor effect [42]. Additionally, a meta-analysis reported that the spliceosome pathway was overexpressed in HCC, but its mechanism of action had not been clarified [43]. To our knowledge, this study firstly reported that CENPL may participate in the occurrence and progress of HCC through these pathways.

Co-expressed genes often have similar functions [24, 44], so we explored the co-expression network of CENPL in HCC through the Coexpedia website. We found that Nuf2, also known as Cell Division Cycle Associated 1 (CDCA1), was the strongest positive correlation co-expressed gene. It was reported that Nuf2 was also highly expressed in HCC and played an important role in the arrangement and correct separation of chromosomes during mitosis [45]. Silencing Nuf2 can induce cell cycle arrest, significantly inhibit HCC proliferation and induce cell apoptosis [46]. Then we performed function and pathway enrichment analysis based on the co-expressed genes and found the biological processes (BP) were also mainly related to cell cycle and metabolism, which were similar to the pathways enriched by CENPL.

With the development of medicine, people gradually realize that traditional TNM staging and pathological grading can only provide limited prognostic information and cannot predict the response to treatment. More and more researchers begin to pay attention to the host's immune system which plays an important role in controlling tumor occurrence and progression, and predicting prognosis and therapeutic response [47]. Previous evidences have shown that tumor progression is often closely related to the decrease of CD8+ T cells, NK cells and other potent lymphocytes that play key anti-tumor roles, and the aggregation of regulatory T cells (Tregs) and tumor-associated macrophages [48–51]. Macrophages can stimulate angiogenesis, enhance tumor cell migration and invasion [52, 53]. This was consistent with the results of our study. Multivariate Cox analysis showed that the infiltration of B cells, CD8+ T cells, and CD4+ T cells were negatively correlated with poor prognosis. That is to say, the decrease of their infiltration indicated an increased risk of poor prognosis. On the contrary, Macrophages were significantly positively associated with poor prognosis, suggesting the high macrophages infiltration predicts adverse prognosis. Elevated CENPL expression could also independently predict worse prognosis under the condition excluding immune cell infiltration. Furthermore, we found that CENPL expression was notably negatively correlated with infiltration of B cells, CD8+ T cells, CD4+ T cells and neutrophil and positively associated with macrophage and dendritic cell, especially had the highest association with macrophages. We speculated that the occurrence and progression of HCC promoted by enhanced CENPL may be partly attributed to augmenting tumorigenic effect of macrophages and attenuating the anti-tumor effect of killer cells such as B cells, CD8+ T cells and CD4+ T cells.

In addition, our results showed that the expression of CENPL was significantly positively correlated with many markers of immune cells, among which IRF5, a marker of M1 macrophages [54], and CCR8 [55] and STAT5B [56], markers of Treg cells, have the highest correlation. Macrophages are often divided into M1 and M2 types according to their functions. Among them, M1 macrophages can secrete inflammatory factors such as interleukin (IL-12), interleukin-6 (IL-6) and tumor necrosis factor alpha (TNFα), promote the production of reactive oxygen species and nitric oxide (NO), and have pro-inflammatory activity [57]. Chronic inflammatory and continuous peroxidation can induce cell cancerization and tumorigenesis. M2 macrophages are involved in accelerating tumor growth, invasion and angiogenesis [52, 53, 57–59]. This study found that the expression of CENPL was positively associated with the markers of M1 and M2 macrophages, but had a higher correlation with M1 macrophages, suggesting that increased CENPL may be more involved in M1 macrophages promoting tumorigenesis. Nevertheless, it may also play a role in the tumor invasion and progression induced by M2 macrophages. Additionally, studies have revealed that Treg cells can inhibit the activity and proliferation of effector CD4+ and CD8+ T cells, and higher Treg cells infiltration means worse prognosis in HCC [49, 50]. This suggests that elevated CENPL may also participate in the process of Treg cells suppressing effector CD4+ and CD8+ T cells. Furthermore, CENPL expression associated prominently and positively with T-cell failure markers such as PD-1, CTLA4, LAG3, TOX, TIGIT, GZMB and Tim-3. Particularly the correlation with Tim-3 was the highest. This explains the result that enhanced CENPL predicts poor prognosis and also provides a basis for finding new immunotherapy methods for HCC patients with poor response to PD-1 antibodies and other immunosuppressive agents.

Tumor immune microenvironment is a complex environment characterized by immunosuppression and immune escape [4, 60]. How to restore the normal anti-tumor immune response to kill tumor cells is a research hotspot in recent years. With the clinical application of immune checkpoint inhibitors (ICIs), new hope has been brought to tumor patients, but only a small number of patients have obtained clinical benefits [4]. Therefore, it is necessary to look for biomarkers to predict the efficacy of ICIs in order to determine the appropriate population for corresponding immunotherapy. Previous studies reported that the expression of PD-L1 and tumor mutational burden (TMB), as biomarkers for the evaluation of ICIs treatment efficacy, could play a better predictive role in some cancers [61–64]. However, other investigators found that PD-L1 and TMB were not significantly associated with ICIs efficacy in most cancer subtypes [65–67]. Therefore, novel biomarkers which can predict the efficacy of ICIs should be developed in combination with PD-L1 and TMB to correctly assess whether patients can benefit from ICIs treatment [66], thereby improving prognosis and reducing unnecessary drug toxicity in patients who are unlikely to benefit. Previous studies have proved that the levels of major markers of immune cells could represent the abundance of corresponding immune cells and the composition of immune cell populations in the tumor-immune microenvironment [68], which were helpful to predict the prognosis and select the best immunotherapy scheme [47, 68–71]. Our study revealed that CENPL expression had a prominent correlation with not only the infiltration level of major immune cells, but also major markers of immune cells. This further verifies our conclusion that elevated CENPL can independently predict poor prognosis of HCC, and to some extent predict the efficacy of some immunotherapy, which provides a direction for new immunotherapy methods.

Many researches provided evidence that almost all tumor types contained abnormal methylation and it could lead to the occurrence of cancer [72, 73]. Studying DNA methylation helps us to understand the mechanism of tumorigenesis and predict the occurrence and progression of cancer [74]. Since methylation is sometimes reversible, it has the potential to become a therapeutic target. Our research unveiled that CENPL had two methylation sites, and CENPL-3′UTR−Open_Sea−cg04555837, one of them, had a significantly positive correlation with shorter survival time.

Although this study is the first to reveal the relationship between CENPL and HCC prognosis and immune infiltration, some false positive rates cannot be ruled out and further experimental and clinical validation is needed.

## CONCLUSIONS

Our study uncovered that elevated CENPL in HCC was positively related to adverse clinicopathological factors, occurrence and progression of HCC, and abnormal immunocyte infiltration. It could be an independent predictor for poor prognosis and a promising determinant for immunotherapy.

## Supplementary Materials

Supplementary Figures
