# Supplementary material for: Highly expressed centromere protein L indicates adverse survival and associates with immune infiltration in hepatocellular carcinoma
Source: Aging (Albany NY). 2021 Oct 4;13(19):22802–29. doi: 10.18632/aging.203574 (PMC8544325; doi:10.18632/aging.203574)
Supplement: Supplementary Figures [file aging-13-203574-s001.pdf]

SUPPLEMENTARY FIGURES

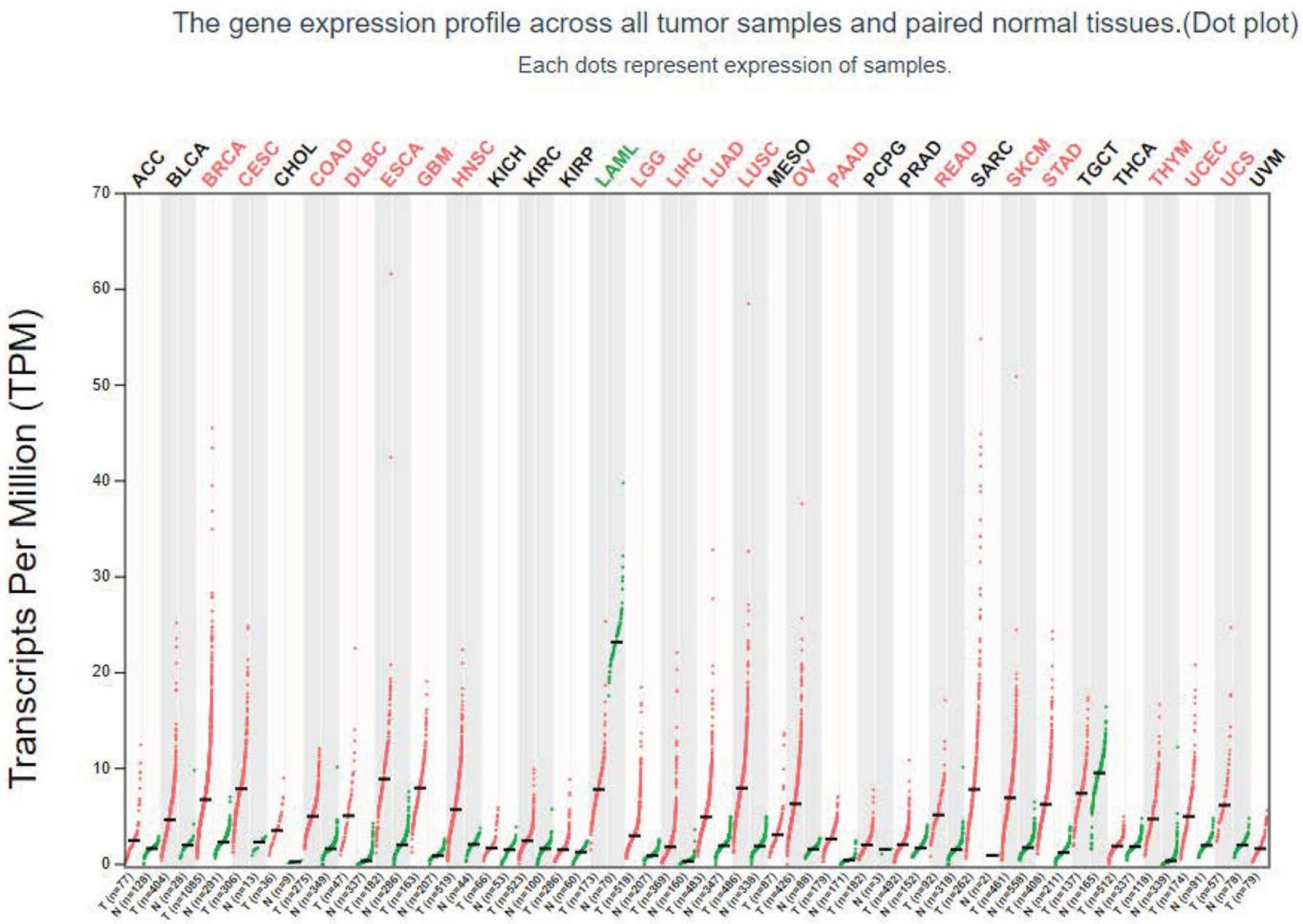

Supplementary Figure 1. The transcription level of CENPL in different tumor types by GEPIA.

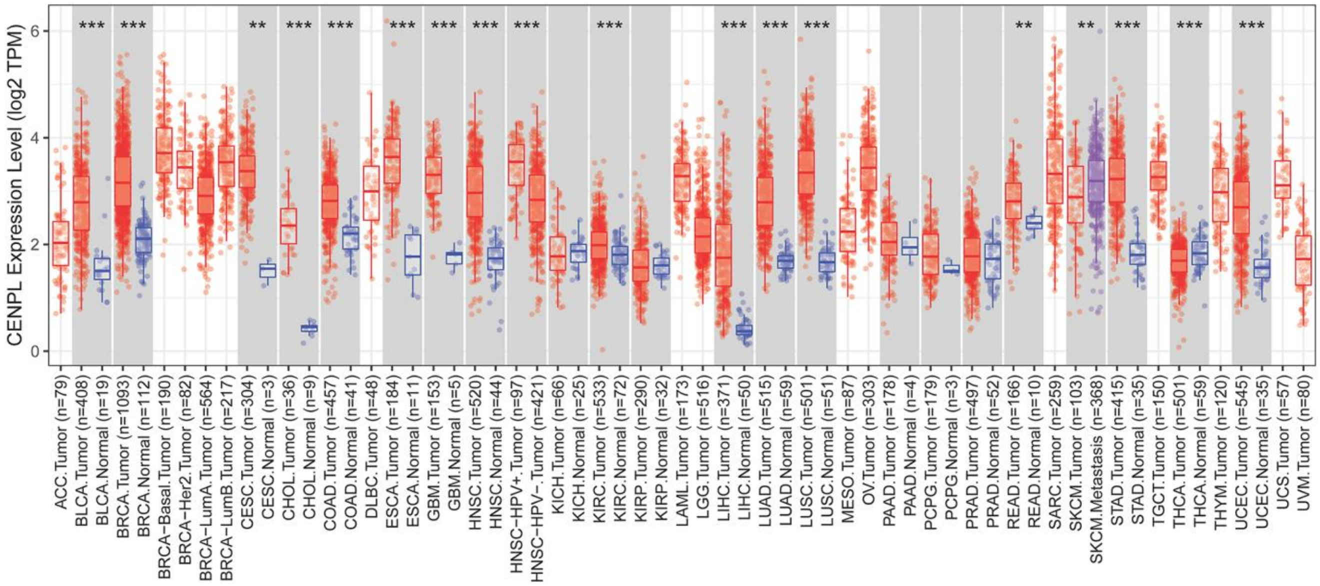

Supplementary Figure 2. The transcription level of CENPL in different tumor types through TIMER.

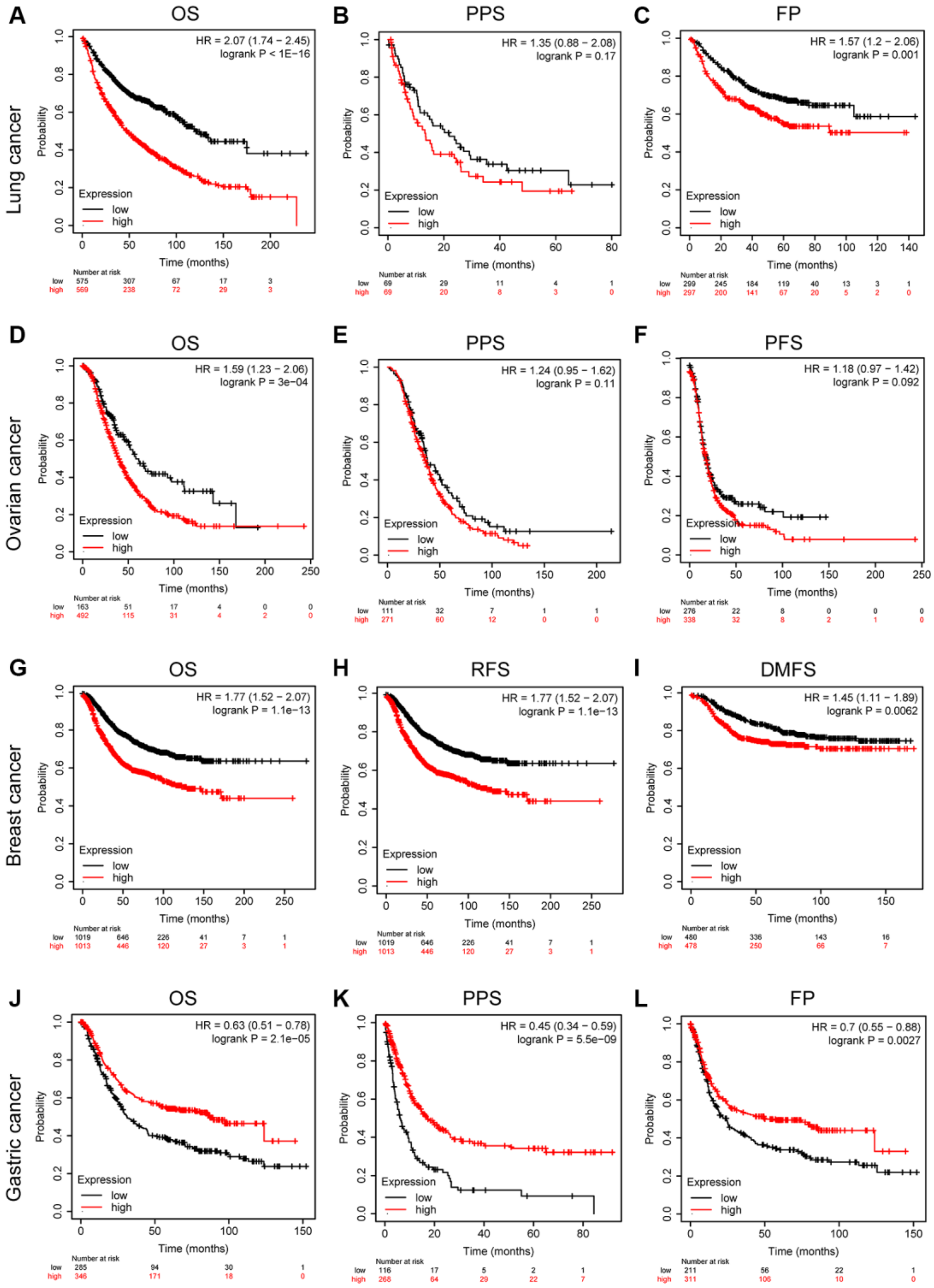

Supplementary Figure 3. The effects of CENPL expression on the prognostic outcome of other tumor types. (**A**–**C**) Lung cancer; (**D**–**F**) Ovarian cancer; (**G**–**I**) Breast cancer, (**J**–**L**) Gastric cancer. Abbreviations: OS: overall survival; PPS: post progression survival; FP: first progression; PFS: progression-free survival; RFS: relapse-free survival; DMFS: distance metastasis free survival.

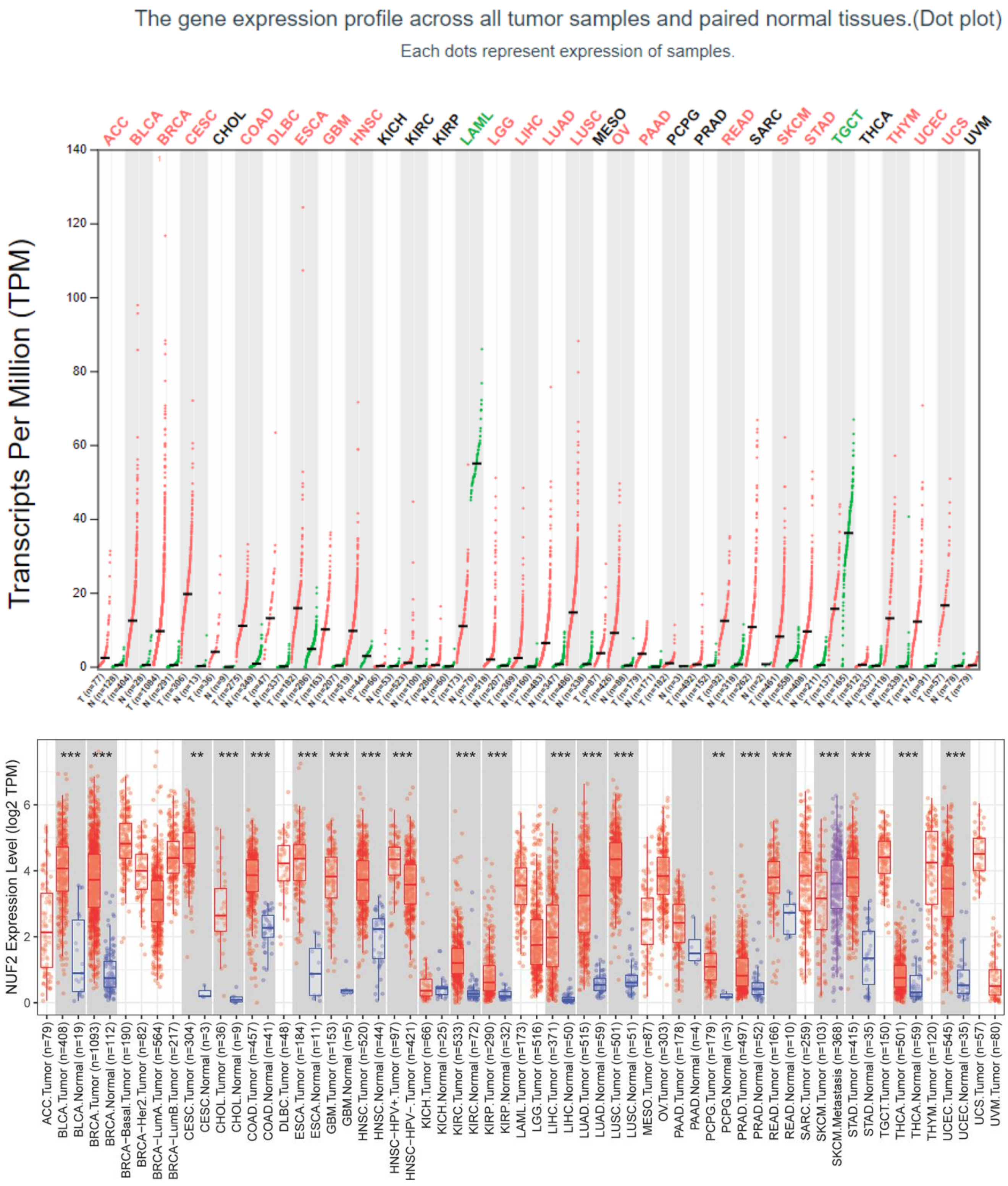

Supplementary Figure 4. The transcription level of Nuf2 in different tumor types through TIMER and GEPIA.

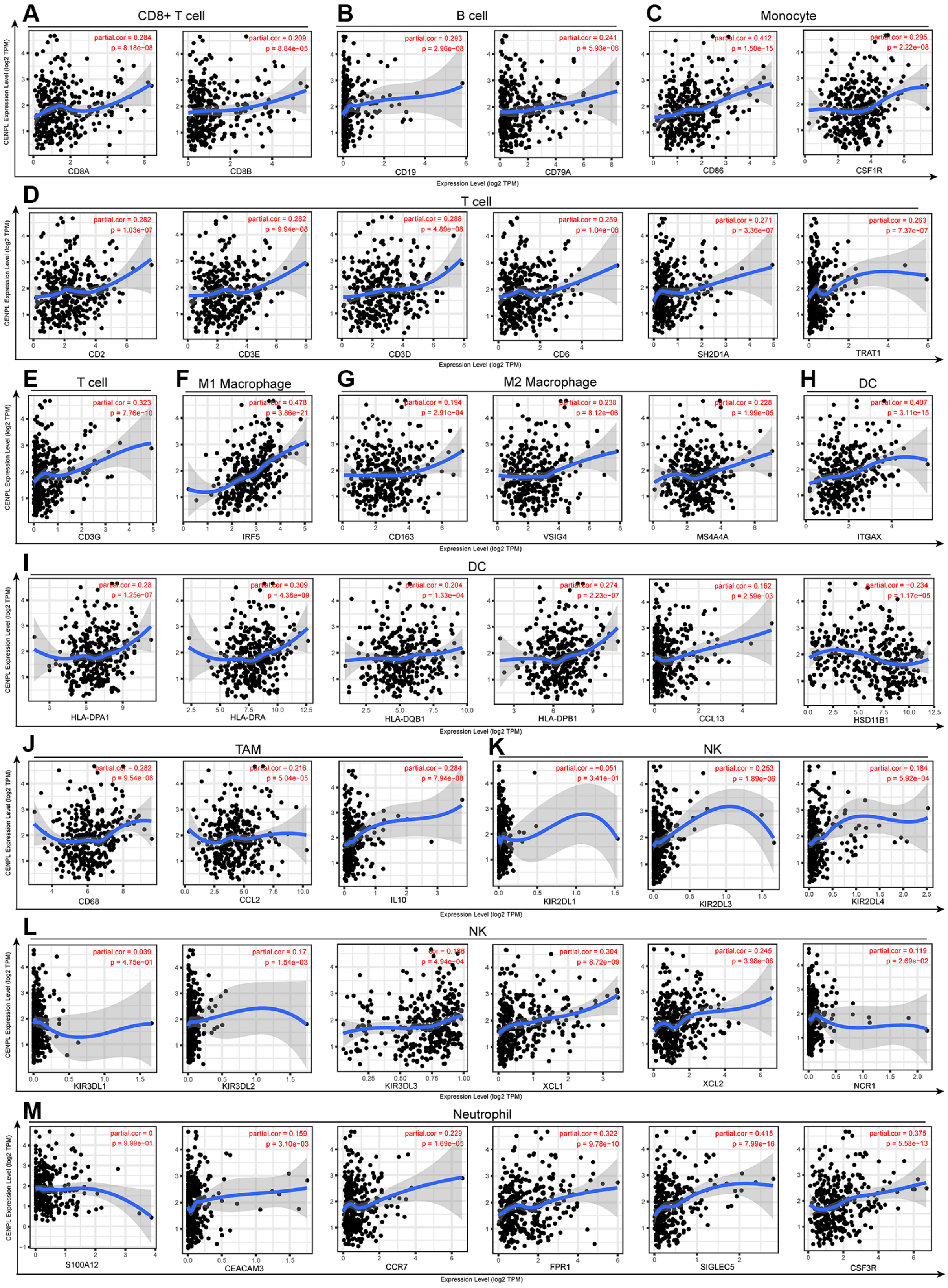
A
CD8+ T cell
partial.cor = 0.284
p = 8.18e−08
CD8A
partial.cor = 0.209
p = 8.84e−05
CD8B
B
B cell
partial.cor = 0.293
p = 2.96e−08
CD19
partial.cor = 0.241
p = 5.93e−06
CD79A
C
Monocyte
partial.cor = 0.412
p = 1.50e−15
CD86
partial.cor = 0.295
p = 2.22e−08
CSF1R
D
T cell
partial.cor = 0.282
p = 1.03e−07
CD2
partial.cor = 0.282
p = 9.94e−08
CD3E
partial.cor = 0.288
p = 4.89e−08
CD3D
partial.cor = 0.259
p = 1.04e−06
CD6
partial.cor = 0.271
p = 3.36e−07
SH2D1A
partial.cor = 0.263
p = 7.37e−07
TRAT1
E
T cell
partial.cor = 0.323
p = 7.76e−10
CD3G
F
M1 Macrophage
partial.cor = 0.478
p = 3.86e−21
IRF5
G
M2 Macrophage
partial.cor = 0.194
p = 2.91e−04
CD163
partial.cor = 0.238
p = 8.12e−06
VSIG4
partial.cor = 0.228
p = 1.99e−05
MS4A4A
H
DC
partial.cor = 0.407
p = 3.11e−15
ITGAX
I
DC
partial.cor = 0.28
p = 1.25e−07
HLA-DPA1
partial.cor = 0.309
p = 4.38e−09
HLA-DRA
partial.cor = 0.204
p = 1.33e−04
HLA-DQB1
partial.cor = 0.274
p = 2.23e−07
HLA-DPB1
partial.cor = 0.162
p = 2.59e−03
CCL13
partial.cor = −0.234
p = 1.17e−05
HSD11B1
J
TAM
partial.cor = 0.282
p = 9.54e−08
CD68
partial.cor = 0.216
p = 5.04e−05
CCL2
partial.cor = 0.284
p = 7.94e−08
IL10
K
NK
partial.cor = −0.051
p = 3.41e−01
KIR2DL1
partial.cor = 0.253
p = 1.89e−06
KIR2DL3
partial.cor = 0.184
p = 5.92e−04
KIR2DL4
L
NK
partial.cor = 0.039
p = 4.75e−01
KIR3DL1
partial.cor = 0.17
p = 1.54e−03
KIR3DL2
cor = 0.186
p = 4.94e−04
KIR3DL3
partial.cor = 0.304
p = 8.72e−09
XCL1
partial.cor = 0.245
p = 3.98e−06
XCL2
partial.cor = 0.119
p = 2.69e−02
NCR1
M
Neutrophil
partial.cor = 0
p = 9.99e−01
S100A12
partial.cor = 0.159
p = 3.10e−03
CEACAM3
partial.cor = 0.229
p = 1.69e−05
CCR7
partial.cor = 0.322
p = 9.78e−10
FPR1
partial.cor = 0.415
p = 7.99e−16
SIGLEC5
partial.cor = 0.375
p = 5.58e−13
CSF3R
CENPL Expression Level (log2 TPM)
Expression Level (log2 TPM)

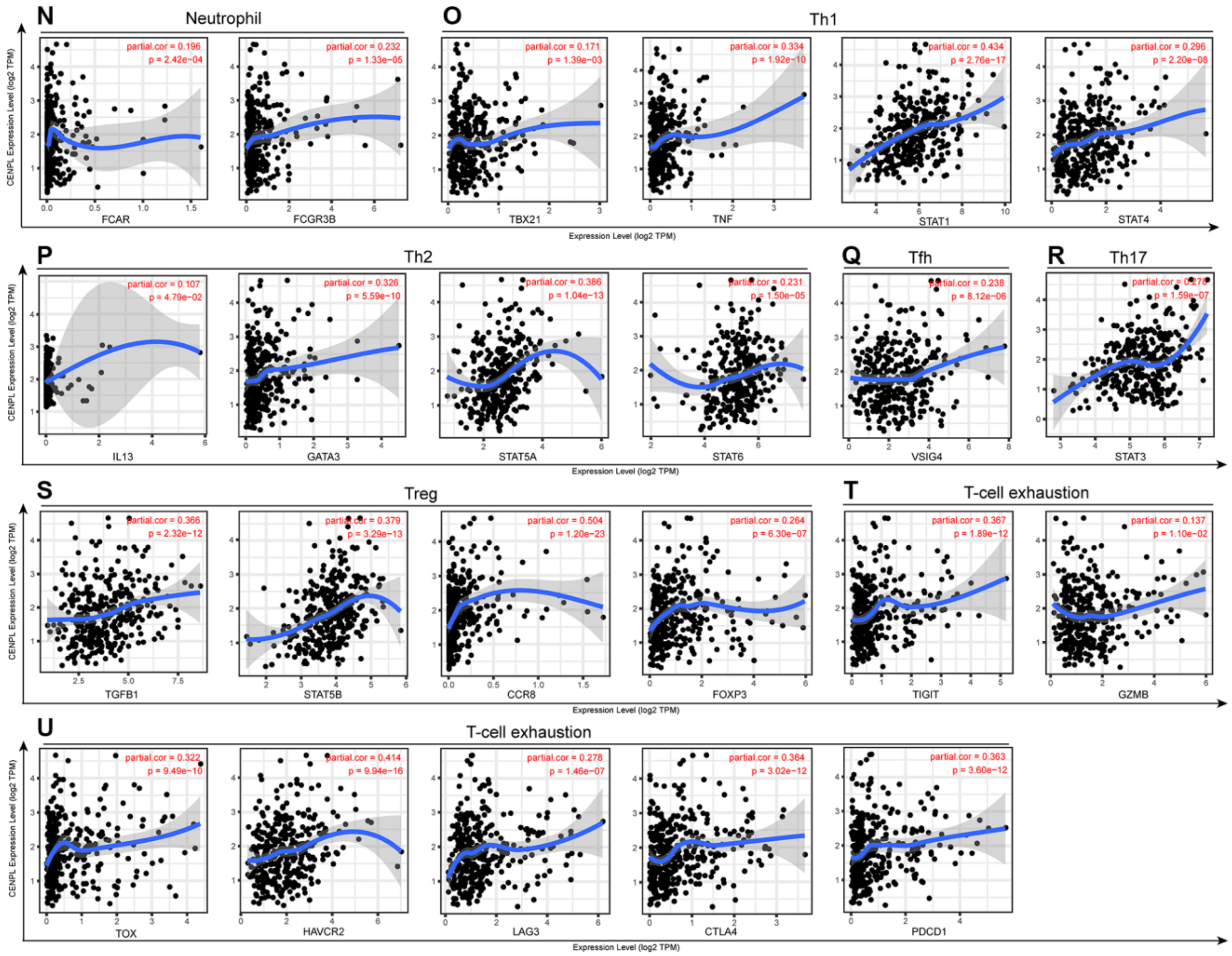

Supplementary Figure 5. Association between CENPL transcription level and immune cell markers. The association between CENPL transcription level and cell markers of (**A**) CD8+ T cell, (**B**) B cell, (**C**) Monocyte, (**D**–**E**) T cell, (**F**) M1 Macrophage, (**G**) M2 Macrophage, (**H**–**I**) DC, (**J**) TAM, (**K**–**L**) NK, (**M**–**N**) Neutrophil, (**O**) Th1, (**P**) Th2, (**Q**) Tfh, (**R**) Th17, (**S**) Treg, and (**T**–**U**) T-cell exhaustion. Abbreviations: DC: dendritic cell; TAM: tumor-associated macrophage; NK: natural killer cell; Th: T helper cell; Tfh: follicular helper T cell; Treg: regulatory T cell.
